# S144. THE ASSOCIATION BETWEEN BRAIN ACTIVITY IN THE PREFRONTAL CORTEX AND DEMOGRAPHIC VARIABLES: A LARGE-SAMPLE FUNCTIONAL NEAR-INFRARED SPECTROSCOPY STUDY

**DOI:** 10.1093/schbul/sby018.931

**Published:** 2018-04-01

**Authors:** Shinsuke Koike, Eisuke Sakakibara, Yoshihiro Satomura, Hanako Sakurada, Mika Yamagishi, Jun Matsuoka, Naohiro Okada, Kiyoto Kasai

**Affiliations:** University of Tokyo

## Abstract

**Background:**

A functional near-infrared spectroscopy (fNIRS) has an advantage of easy measurement of the activity in the surface of the cortex with a naturalistic position. Therefore, fNIRS has been used as an aid for differential diagnosis of depressive symptoms as a clinical application in Japan. However, the fNIRS diagnosis system is not considered gender, age, and task performance which could be associated with brain activity. We previously reported that the fNIRS brain activity was associated with gender, age, cognitive performance, age at onset, and clinical stages of psychosis. Therefore, we intend to explore the association between fNIRS brain activity in the prefrontal cortex and demographic variables using a large sample size.

**Methods:**

Of 163 patients with schizophrenia and 470 healthy controls who were measured using a fNIRS instrument from April 2004 to April 2016, 224 measurements from 152 patients and 475 from 386 controls were analyzed after exclusion by the criteria. We analyzed the intensity and timing of brain activity during the letter version of a verbal fluency task in the subregion of the prefrontal cortex.

The associations between brain activity and demographic variables were tested using general linear mixed models with the main effect of gender, age, group and interaction by group as fixed effects, and measurement time and interval by participant as random effects. We compared the models including all possible combination of the fixed effects. Then we further tested the association between brain activity and measurement time, measurement interval, task performance, sleepness, premorbid IQ, handedness, and education year by adding the main effect of each variable and interaction by group into the best-fitted model.

**Results:**

Model comparison showed that the best fitted and reliable model included the main effects of gender, age, and group for the intensity of brain activity in the prefrontal cortex. The intensity was smaller when female, older, and schizophrenia group. The best model for the timing included main effects of group and task performance, showing the timing was earlier when control group and better task performance.

**Discussion:**

To the best of our knowledge, this is the first study which investigated the association between brain activity and demographic variables in a large sample set assessed by the same instrument and task. In future, the improvement of the clinical application fNIRS system adding to demographic variables is needed.

